# Artificial Intelligence-Related Risks in Interventional Pulmonology: An Exploratory Enumeration and Ranking Study Across Five General-Purpose Large Language Models

**DOI:** 10.3390/jcm15197340

**Published:** 2026-09-22

**Authors:** Guido Marchi, Lorenzo Corbetta

**Affiliations:** 1 Pulmonology Unit, Cardiothoracic and Vascular Department, University Hospital of Pisa, Via Paradisa 2, 56124 Pisa, Italy; 2 Department of Experimental and Clinical Medicine, University of Florence, Viale Morgagni 63, 50134 Florence, Italy; lorenzo.corbetta@unifi.it

**Keywords:** artificial intelligence, large language models, interventional pulmonology, bronchoscopy, risk assessment, automation bias, clinical decision support, deskilling, patient safety

## Abstract

**Background/Objectives:** Artificial intelligence (AI) is entering interventional pulmonology (IP) faster than its potential risks have been systematically catalogued. We explored whether general-purpose large language models (LLMs), now widely consulted informally by patients and clinicians, could provide a rapid and structured means of enumerating and ranking candidate AI-related risks in IP, potentially contributing to risk awareness and hypothesis generation. **Methods:** Five LLMs (ChatGPT, Claude, Gemini, Grok, DeepSeek) were each queried once, in independent, memory-free sessions, with one standardised prompt requesting ten ranked AI-related risks with impact and likelihood scores (1–5); an informal repeat administration was performed, but output stability was not formally assessed. The resulting 50 risk statements were inductively coded, by an AI coder with independent human validation by two reviewers, into 15 constructs nested in 8 higher-order domains. **Results:** Mean self-assigned impact was 3.92 (SD 0.78) and mean likelihood 3.54 (SD 0.68). The eight domains comprised AI technical/perceptual accuracy (diagnostic, detection and navigational error); automation bias and over-reliance; generalisability, algorithmic bias and health equity; model and system reliability over time; erosion of procedural competence; explainability, transparency and accountability; cybersecurity, privacy and data integrity; and cognitive and workflow burden. Five domains were raised by all five models and the remaining three by four of five. Two domains-AI technical/perceptual accuracy and automation bias-together accounted for every model’s two highest-ranked risks, and no risk statement outside these two domains was ranked first or second by any model. Deskilling (erosion of procedural competence), although listed by all five models, was never ranked above third by any model, whereas practising IP specialists in our prior international survey rated it the single highest research priority. **Conclusions:** These exploratory and hypothesis-generating findings suggest that general-purpose LLMs may have potential as a rapid, low-burden means of enumerating and ranking candidate AI-related risks and broadening awareness of issues that may warrant further investigation in IP. The lower ranking of deskilling by the LLMs than by domain experts indicates that LLM-generated rankings may under-prioritise risks that clinicians consider most important. Although their outputs are not validated measures of clinical risk and do not replace expert appraisal, we believe they may provide a starting point for subsequent human-led risk assessment and prioritization in an area of research that remains largely unexplored yet is highly relevant to patient safety.

## 1. Introduction

Artificial intelligence (AI) is being integrated into interventional pulmonology (IP) across an expanding range of applications, including AI-assisted pulmonary nodule detection and radiomic risk stratification [1,2,3,4], robotic and computer-aided bronchoscopic navigation [5], AI-enhanced endobronchial ultrasound (EBUS) interpretation and bronchoscopy more broadly, decision support for rapid on-site evaluation, and automated analysis of thoracic ultrasound and imaging for pleural disease [6,7,8,9,10,11]. These technologies are widely anticipated to improve diagnostic yield, procedural precision, and access to advanced bronchoscopic care [6,7,8,12]. Comparable developments are under way across clinical, surgical, and laboratory medicine [13], including AI-supported screen reading in mammography screening, evaluated in a randomised controlled trial [14]; real-time computer-aided polyp detection during colonoscopy, an invasive endoscopic procedure in which a meta-analysis of randomised trials showed higher adenoma detection rates [15]; computer vision and clinical decision support in surgery [16]; and machine learning-based applications in laboratory medicine [17]. As in other areas of medicine, however, the pace of clinical integration is outstripping the pace at which the associated risks are being systematically characterised [18,19,20].

A parallel and increasingly consequential development is the rapid, informal adoption of general-purpose conversational AI systems—large language models (LLMs) such as ChatGPT, Claude, Gemini, Grok, and DeepSeek—by clinicians, trainees, and patients as an ad hoc source of medical information, second opinions, and even ethical or risk-related reasoning [21,22]. A recent large-scale review characterised the safety and security landscape of LLMs in healthcare broadly, cataloguing failure modes spanning hallucination, bias, privacy, and inappropriate over-reliance [23,24]. Whether such general-purpose systems, precisely because they are trained on vast corpora that include the biomedical, regulatory, human-factors, and AI-safety literature, could also be turned into a structured elicitation instrument—way of rapidly surfacing candidate risk domains for a specific clinical subspecialty—has not, to our knowledge, been explored for IP, although a general-purpose LLM has been used to generate candidate research priorities in gastroenterology that were subsequently rated by experts [25].

One specific risk that has received growing dedicated attention is AI-induced deskilling: the erosion of previously acquired clinical competencies through reduced independent practice consequent to automation, driven by the interacting mechanisms of automation bias [26], cognitive offloading, and upskilling inhibition in trainees [27,28,29,30,31]. The first prospective clinical evidence for this phenomenon came from colonoscopy, where sustained exposure to AI assistance was associated with a significant subsequent decline in unassisted adenoma detection [32]. The applicability of the European Union (EU) Artificial Intelligence Act [33] to this phenomenon has itself become a subject of active legal-ethical debate [34]. We recently reported the first international survey of IP specialists’ perceptions of AI-induced deskilling, in which 118 practitioners from ten countries expressed substantial concern about procedural skill erosion (73%) and upskilling inhibition (83%), and rated deskilling the single highest-priority item for future research among twelve surveyed constructs (89%) [35]. That survey, like the deskilling literature more broadly, necessarily focused on one risk domain in depth. It did not attempt to situate deskilling within the broader landscape of AI-related risk that IP as a specialty may face, nor did it explore whether other, less-studied risk domains might be equally or more salient.

The present study takes a deliberately different and complementary approach. Rather than surveying human experts about a pre-specified risk domain, we used five widely available general-purpose LLMs as parallel, separately queried “informants,” each asked with one standardised, structured prompt to generate and rank ten AI-related risks specific to IP, together with a description, presumed mechanism, affected stakeholders, impact and likelihood ratings, and a mitigation strategy for each. This design is intentionally exploratory and hypothesis-generating rather than confirmatory. We do not claim that LLM-generated risk statements constitute validated empirical evidence of real-world harm, nor do we compare the five models against one another in terms of output quality or clinical accuracy; the AI systems are used here purely as a content-generation and pattern-elicitation method, analogous in spirit to using a structured brainstorming or horizon-scanning technique, whose output is then treated as raw qualitative data for inductive analysis—not as a source of clinical fact.

The aims of this study were therefore: (1) to elicit, from five general-purpose LLMs, a structured set of AI-related risk statements for IP using one standardised prompt; (2) to inductively synthesise the resulting statements into a taxonomy of constructs and higher-order domains, and to describe convergence and divergence across models; (3) to compute descriptive and exploratory statistics on the collected impact, likelihood, and rank data; and (4) to situate the resulting risk landscape, narratively and non-statistically, against our own previously published human-expert survey findings on AI-induced deskilling in IP [35], as a minor point of comparison. We report this work as an original, hypothesis-generating exploratory study, consistent with the emerging-technology, low-prior-evidence context in which it is conducted.

## 2. Materials and Methods

### 2.1. Study Design

This was an exploratory, qualitative, multimodel content-elicitation study. No human participants, patient data, or animal data were involved at any stage; the units of analysis were text outputs generated by five commercially available, general-purpose conversational AI systems in response to a single standardised prompt. The study was not pre-registered, consistent with its exploratory, hypothesis-generating purpose, and no formal a priori sample size calculation applies, since the “sample” is the fixed, complete set of outputs from five pre-specified AI systems rather than a probabilistic sample of a larger population.

### 2.2. Selection of AI Systems

Five general-purpose LLM-based conversational AI systems were selected on the basis of being, at the time of the study (August 2026), among the most widely deployed publicly available systems from five different developers: GPT-5.6 Luna (OpenAI, San Francisco, CA, USA), Claude Sonnet 5 and Claude Opus 5 (Anthropic, San Francisco, CA, USA), Gemini 3.1 Pro (Google, Mountain View, CA, USA), Grok 4.5 (xAI, Palo Alto, CA, USA), DeepSeek-R1 (DeepSeek, Hangzhou, China). The inclusion criterion was developer and platform diversity rather than benchmark performance ranking, since the purpose of the study was to sample broadly across the general-purpose LLM ecosystem that clinicians might plausibly encounter, rather than to identify the single best-performing model. Table 1 summarises the specific model version, reasoning/thinking mode, platform, and session conditions for each system, as recorded by the operator (G.M.) at the time of data collection.

### 2.3. Standardised Prompt and Administration Protocol

A single standardised prompt (verbatim text provided as Supplementary Table S1) was developed specifying: the clinical context (interventional pulmonology); a request for exactly ten AI-related risks, ranked from highest to lowest overall priority; and, for each risk, a required structured format comprising a risk title, description, presumed mechanism, affected stakeholder(s), an impact rating (1–5), a likelihood rating (1–5), and a mitigation strategy. No example risks, scoring anchors, or prior model outputs were provided in the prompt, so as not to bias content generation toward any pre-specified taxonomy; the eventual taxonomy (Section 2.5) was derived inductively from the outputs, not imposed on the prompt. Ten risks were requested from each model as a pragmatic compromise between breadth and depth: a list of this length extends beyond the few most obvious risks while keeping a fully structured, seven-field output for every item within a single response, and it provides an identical, fixed denominator across models for the convergence analysis and a sufficient number of ranked items for the within-model self-consistency analysis (Section 2.6). A ten-item format is also used, for example, in the annual Top 10 Health Technology Hazards list published by ECRI [22]. The ten risks requested per model should not be confused with the eight higher-order domains reported in the Results, which were not requested from the models but were derived inductively from their outputs (Section 2.5).

Each of the five systems was queried entirely independently, in a fresh session configured to exclude conversational memory and prior chat history where the platform offered such a mode (temporary/incognito chat; no prior session context), so that each output would reflect the model’s default response to the prompt in isolation rather than being shaped by an ongoing conversation. All sessions were conducted by the same operator (G.M.), via a desktop web browser, on 21 August 2026 (Table 1). Web search/browsing functions and other external tools were not specifically disabled: each system was used through its standard web interface with the default platform settings in force at the time of data collection, apart from the reasoning mode and temporary-chat configuration reported in Table 1. Because any use of real-time web retrieval during response generation was not systematically documented, a contribution of retrieved web content to individual outputs cannot be excluded; these access conditions are reported in line with current recommendations for the transparent reporting of studies using LLMs [36]. As an informal, real-time consistency check [37], the prompt was administered twice to each model, in two independent fresh sessions; the operator compared the two outputs at the time of collection and judged that broadly similar risk themes recurred in both administrations for all five models. For each model, the complete structured output from one administration—the full ten-row risk table, the model’s own free-text priority-ranking rationale, and a screenshot of the session—was retained as the analysed record; the fifty resulting risk statements (five models × ten ranked risks) constitute the dataset analysed in this study. Because only one administration per model was retained as a complete, verbatim, structured transcript, a formal quantitative stability statistic (e.g., a rank-order correlation between the two runs) could not be computed, and the consistency check reported here is therefore an unquantified operator observation at the time of collection, not an independently archived and re-analysed second dataset. Accordingly, output stability was not formally assessed in this study and no claim of demonstrated stability is made, particularly as repeated administration of an identical prompt to the same LLM can yield non-identical outputs [37,38].

### 2.4. Data Extraction

All five structured outputs were transcribed into a single dataset comprising, for each of the 50 risk statements: the source model, the model-assigned rank (1–10), the verbatim risk title, impact score (1–5), likelihood score (1–5), and the verbatim description, mechanism, affected-stakeholder, and mitigation text. No wording, score, or rank was altered, corrected, or re-ordered by the authors at any stage; the dataset is reproduced in full in Supplementary Table S2.

### 2.5. Qualitative Coding

The 50 risk statements were inductively coded into a two-level scheme: 15 first-order constructs, grouped into 8 higher-order domains, developed from the data with no pre-existing taxonomy imposed [39]. Coding proceeded by (i) reading all 50 statements’ titles, descriptions and mechanisms; (ii) grouping statements describing substantively the same underlying phenomenon into constructs; (iii) grouping related constructs into higher-order domains; and (iv) assigning each of the 50 statements a single primary domain and construct code based on its stated title and mechanism, to keep the quantitative summary (Section 2.6) unambiguous. Where a statement’s title or mechanism explicitly spanned two constructs (as occurred for four of the fifty statements, each combining two adjacent concepts into one item, e.g., “AI-driven diagnostic or navigational error”), this is noted narratively and the secondary construct is cross-referenced, but only the primary code contributes to the counts reported in Results. The full coding matrix, including construct-level definitions and item-by-item assignments, is provided in Supplementary Table S2. The constructs and domains were derived through the inductive qualitative coding procedure described above, performed by a generative AI coder under human review, and not by a clustering or other computational integration algorithm: Python was used exclusively for the descriptive statistics described in Section 2.6. Consequently, the domains represent pragmatic, data-driven groupings of the concepts generated by the models, not a theory-derived or logically exhaustive framework. Some domains group conceptually distinct but related aspects; for example, the domain combining generalisability, algorithmic bias, and health equity (D3; Section 3.3) reflects the close link between these aspects, since inequitable model performance commonly arises from development data or labels that do not adequately represent the populations, settings, or devices in which a model is deployed [24,40]. A finer-grained, layered view is provided by the 15 constructs and by the item-level assignments in Supplementary Table S2. Although generative AI is increasingly used to support qualitative coding, its use requires comparison with human coding and human oversight [41].

Coding was performed by a single coder: a generative AI system (Claude, Anthropic), operating under the direct supervision of, and with all coding decisions reviewed and approved by, the corresponding author (G.M.). Independent human validation of the complete coding was subsequently performed by two reviewers, so that the final assignment of each risk statement to its construct and domain does not rest on the AI coder’s classification alone. Because the model used as the coder (Claude Sonnet 5) also generated one of the five analysed outputs, this validation step mitigates the associated risk of circularity (Section 4.10). In accordance with the journal’s policy on generative artificial intelligence (GenAI), the authors disclose the following uses. Beyond the five LLM outputs that constitute the primary data of this study (Section 2.2, Section 2.3 and Section 2.4), Claude Sonnet 5 (Anthropic) was used as the single coder for the qualitative coding described above, under the direct supervision and final approval of the corresponding author (G.M.); GPT-5.6 Luna (OpenAI) was used for language editing and for generating the graphical layout of Figure 1; and, during the revision of the manuscript, Claude Opus 5 (Anthropic) was used to assist with drafting revised text in response to the reviewers’ comments and with identifying candidate references, each of which was checked against PubMed records, publisher websites, or official sources before inclusion. No GenAI tool was used to alter, select, or interpret the recorded impact, likelihood, or rank data, and all statistical analyses were performed by the authors (Section 2.6). Product and version details are reported in the Acknowledgments; the authors have reviewed and edited all AI-assisted output and take full responsibility for the content of this publication.

### 2.6. Statistical Analysis

All statistical analysis was descriptive or exploratory in nature; no formal inferential hypothesis testing was performed, consistent with the non-probabilistic, fixed, five-system design. Because impact and likelihood were self-assigned by the models on non-anchored 1–5 ordinal scales, they were treated as ordinal data; means and standard deviations, overall and per model, are reported only as compact descriptive summaries to facilitate comparison across models and domains, a common but debated practice for Likert-type ratings [42,43], and should not be interpreted as interval-level measurements. The association between impact and likelihood scores across all 50 items was described with Spearman’s rank correlation coefficient (ρ), a rank-based measure appropriate for ordinal data. Within each model, the Spearman rank correlation between the model’s stated rank position (1–10) and the product of its own impact and likelihood scores was computed as an exploratory self-consistency check (i.e., whether each model’s stated priority order tracked its own severity/likelihood scoring); given *n* = 10 per model, these are reported as descriptive/exploratory associations, not as confirmatory hypothesis tests. Because the five systems constitute a fixed, non-probabilistic set rather than a random sample from a defined population, *p*-values have no inferential interpretation in this design and are not reported; all correlation coefficients are presented as descriptive measures only. Domain- and construct-level convergence across models is reported as the number of models (of five) contributing at least one primary-coded item to each domain/construct. All computations were performed in Python 3 (SciPy, pandas, NumPy); the analysis script and its full console output are provided as Supplementary Material (File S1) for transparency and reproducibility.

### 2.7. Comparison with Prior Human-Expert Survey Data

As a minor, exploratory point of discussion, selected findings were juxtaposed narratively against previously published data from the authors’ own international cross-sectional survey of 118 IP specialists’ perceptions of AI-induced deskilling [35]. This comparison is descriptive and non-statistical: the two studies differ fundamentally in design (structured Likert-scale survey of human experts vs. open-ranked elicitation from AI systems), sampling unit, and instrument, and no formal statistical test of concordance between them is valid or was attempted. The comparison is offered solely to illustrate points of qualitative convergence and divergence between AI-generated and human-expert risk framing, as a hypothesis-generating observation for future research.

## 3. Results

### 3.1. Overview of Collected Outputs

All five AI systems returned a complete, correctly formatted set of ten ranked risk statements in response to the standardised prompt, with no missing fields; no re-querying or prompt modification was required for any model. All sessions were conducted on 21 August 2026, each in a temporary/incognito session with no memory or prior conversation history (Table 1). The resulting dataset comprised 50 risk statements in total.

### 3.2. Descriptive Statistics of Impact and Likelihood Ratings

Across all 50 risk statements, the mean self-assigned impact score was 3.92 (SD 0.78; range 3–5) and the mean likelihood score was 3.54 (SD 0.68; range 2–5). Impact and likelihood scores were not meaningfully correlated across the pooled dataset (Spearman ρ = −0.16), indicating that models did not simply equate “more severe” with “more likely,” or vice versa, when assigning these two ratings.

Per-model mean ratings are reported in Table 2. Mean impact ranged from 3.50 (Claude) to 4.40 (ChatGPT); mean likelihood ranged from 3.30 (Claude) to 3.70 (Gemini). Within every individual model, the Spearman correlation between the model’s stated rank position and its own impact × likelihood product was strong and in the expected direction (ρ from −0.86 to −0.93; *n* = 10 per model), indicating that each model’s self-reported priority order was internally consistent with the severity and likelihood scores it had itself assigned. Given the small per-model sample (*n* = 10), this is reported as a descriptive self-consistency observation rather than a confirmatory statistical test, and should not be read as evidence of external or clinical validity.

### 3.3. Qualitative Taxonomy: Domains and Constructs

Inductive coding of the 50 risk statements produced 15 first-order constructs, nested within 8 higher-order domains (full definitions and item-level coding in Supplementary Table S2; schematic overview in Figure 1). Table 3 summarises, for each domain, the number of contributing statements, the number of models (of five) contributing at least one statement, and the mean impact and likelihood scores of its constituent statements.

Five of the eight domains—D1 (AI technical/perceptual accuracy: diagnostic/interpretive and navigational/targeting error), D2 (automation bias and over-reliance), D3 (generalisability, algorithmic bias and health equity), D5 (deskilling and erosion of procedural competence), and D6 (explainability, transparency and accountability)—were raised by all five models. The remaining three domains—D4 (model and system reliability over time), D7 (cybersecurity, privacy and data integrity), and D8 (cognitive/workflow burden)—were each raised by four of five models: D4 was the only domain not represented in DeepSeek’s output, while D7 and D8 were the only domains not represented in Claude’s output. Claude was the only model with two entirely absent domains; every other between-model gap involved a single model and a single domain.

Two domain-level severity profiles are noteworthy. D7 (cybersecurity) had the highest mean impact of any domain (4.50) but the lowest mean likelihood (2.75), a high-severity/lower-probability profile consistent with conventional security-risk framing [44]. D8 (alert fatigue) had the lowest mean impact of any domain (3.00) [45]. D6 (explainability/accountability), despite being raised by all five models with the largest number of constituent statements jointly with D1 (nine each), consistently occupied the latest average priority position among the universally raised domains: the single best (lowest-numbered) rank any model assigned to a D6 item was rank 5 (DeepSeek), with the other four models’ best D6 item ranked 6th or 9th. D6 was thus the domain that was simultaneously the most consistently recognised across models and the most consistently deprioritised.

### 3.4. Concentration of Top-Ranked Risks

Across the five models’ combined top-2-ranked risks (ten statements: each model’s rank 1 and rank 2), all ten fell within just two domains and three constructs. Six of ten top-2 statements were coded to D1 (technical/perceptual accuracy: three diagnostic/interpretive, three navigational/targeting), and four of ten to D2 (automation bias). Considered separately, four of five models’ single top-ranked (rank 1) risk was a D1 item; the exception was DeepSeek, whose rank-1 item was titled and mechanistically framed around automation bias (D2) but whose stated clinical outcome—missed malignancy—is diagnostic in nature, illustrating the close conceptual coupling between these two domains even where they were coded separately. At rank 2, the pattern inverted: three of five models’ rank-2 item was a D2 (automation bias) statement, and two of five were D1 (navigational) statements. No risk statement outside D1 or D2 was ranked first or second by any model.

## 4. Discussion

### 4.1. Principal Findings

Five general-purpose LLMs, queried independently with one standardised prompt and no coordination or shared context between sessions, converged on the same two priority domains—AI technical/perceptual accuracy (diagnostic/interpretive and navigational error) and automation bias/over-reliance—for every one of their combined top-two-ranked risks. Five of eight inductively derived domains were raised by all five models, indicating a broad core of shared risk framing; at the same time, two domains were entirely absent from one model’s output (cybersecurity and alert fatigue, both absent from Claude) and one domain was absent from another’s (model/system reliability, absent from DeepSeek), indicating that no single model’s spontaneous output reproduced the full breadth of risk domains that the five-model set jointly generated. Deskilling, although raised by all five models, was never ranked above third priority by any of them—a pattern that stands in descriptive contrast to our own prior survey finding that deskilling was the single highest-priority research topic identified by 118 practising IP specialists [35].

### 4.2. Convergence on Technical Accuracy and Automation Bias

The predominance of diagnostic/navigational accuracy and automation bias among the top-ranked risks across all five models is consistent with what is structurally distinctive about IP among AI-augmented procedural fields: image-guided and robotic-assisted bronchoscopy require continuous, real-time integration of AI-derived spatial and interpretive information directly within the perception–action loop of the procedure itself, rather than the retrospective or asynchronous review of AI outputs that characterises radiology or histopathology reporting [35]. Errors of the kind captured in our D1 domain (mislocalised lesions, registration drift, misclassified tissue) or over-trust of the kind captured in D2 (automation bias) are therefore plausibly positioned, by any reasoner—human or model—as more directly and immediately consequential in IP than they might be in a specialty where a clinician retains full independent control of the primary diagnostic task [7,8]. The consistency with which five separately queried models from different developers converged on this same pairing, without having been prompted with any risk taxonomy or example, is a striking, if exploratory, signal—though it should be read as evidence about the structure of publicly available AI-safety discourse concerning image-guided procedural AI, which these models were trained on, rather than as independent empirical confirmation that these are in fact the two most consequential real-world risks in IP. Because the training corpora of general-purpose LLMs are likely to overlap substantially, the five systems should not be regarded as fully independent observers, and cross-model convergence may reflect shared underlying literature and public discourse rather than independent confirmation of a risk domain (Section 4.8).

### 4.3. Automation Bias: A Possible Educational Complementarity

All five models spontaneously generated “automation bias” or a close conceptual equivalent (“over-trust in algorithmic outputs”) as a top-tier construct, without this term or concept being present anywhere in the prompt. This is notable when set against our prior survey finding that only 38% of practising IP specialists reported prior familiarity with automation bias as a named concept, even though 81% recognised its clinical relevance once a standardised definition was provided—a 43-percentage-point gap interpreted there as reflecting a conceptual-literacy deficit rather than a lack of intuitive concern [35]. The readiness with which general-purpose LLMs surface this specific, well-established human-factors construct raises the hypothesis, to be tested rather than assumed, that structured LLM-assisted elicitation exercises of the kind used here could have some value as an adjunct to curriculum design—for example, as a low-cost way of surfacing established terminology and framing for concepts that practising clinicians recognise operationally but may not yet have named. We advance this only as a hypothesis; no educational intervention or outcome was tested in this study.

### 4.4. Deskilling: Convergent in Kind, Divergent in Priority

Every model in this study identified deskilling as a risk domain, echoing a rapidly growing dedicated literature [27,28,29,30,31,32]. Yet in a competitive, ten-item, forced-ranking task, no model placed deskilling above third priority (best rank achieved: Gemini, rank 3; mean best rank across the five models, 4.8), and it was the concern that, on average, appeared latest among the universally recognised domains behind D1, D2 and D3. This can be juxtaposed, descriptively and without any statistical test of concordance, against our prior survey, in which deskilling was rated the single highest research priority by human IP specialists among twelve explicitly probed constructs (89% agreement) [35]. We regard this divergence as one of the most important findings of the present study. The risk that practising IP specialists had rated as their highest research priority was never ranked above third by any of the five LLMs, suggesting that LLM-based enumeration and ranking, which reflects the published discourse on which the models were trained (Section 4.8), may under-prioritise risks whose importance is most evident to clinicians with first-hand procedural and training experience. This divergence reinforces the need for domain-expert input when AI-related risks in IP are prioritised, and it supports giving deskilling explicit attention in IP training, credentialing, and research agendas, rather than inferring its relative importance from LLM-derived rankings.

We nevertheless avoid over-interpreting this contrast as definitive evidence that LLMs “underrate” deskilling, for at least two reasons that must temper any such claim. First, the two instruments measured different things: the survey asked human respondents to rate their level of agreement with a single, explicitly named deskilling-related statement in isolation, whereas the present study asked LLMs to generate and rank deskilling in open competition against nine other self-generated risk types—a considerably more demanding salience test that any risk domain, however important, might fail to top. Second, the populations are not comparable: the survey respondents were practising clinicians personally embedded in procedural training pipelines and mentorship structures, for whom skill acquisition and maintenance may carry a felt, first-person salience that a general-purpose LLM’s training-data-derived synthesis of public discourse cannot be assumed to reproduce. What the two datasets can jointly support is a more modest observation: deskilling is a risk domain on which practising IP specialists and general-purpose LLMs agree in kind (both identify it as a relevant risk) but not in relative priority when forced to rank it against other AI-related harms—a divergence that itself seems worth further, purpose-designed investigation rather than resolution here.

### 4.5. Between-Model Differences in Risk Framing

Beyond the domains that converged across all five models, two domains were each missing from one model’s spontaneous top-ten list: cybersecurity/data integrity and cognitive/workflow burden were both absent from Claude’s output, and model/system reliability over time was absent from DeepSeek’s. No inference about any model’s general capability or safety orientation should be drawn from this—the task was an unconstrained, forced-choice, ten-item ranking, so any risk type not selected by a given model may simply have been judged lower priority by that model on that occasion, not unknown to it. The practical implication we would draw is methodological rather than evaluative: a single LLM’s spontaneous output, taken alone, may not span the full breadth of risk domains that a multimodel approach can surface, which is consistent with a broader rationale for using several separately queried AI “informants”—or, in a clinical context, for not relying on any single AI system’s own risk framing as a complete account of its own limitations.

### 4.6. Relation to the Broader LLM-Safety-in-Healthcare Literature

Our IP-specific, procedurally grounded top risks—technical/perceptual accuracy and automation bias—sit within, and are broadly consistent with, the wider taxonomy of LLM-related harms recently catalogued for healthcare in general, which spans hallucination and factual error, algorithmic and representational bias, privacy and security failure, and inappropriate clinical over-reliance [23]. What the present exploratory study adds is a subspecialty-specific, empirically elicited (rather than expert-panel-derived) ranking, suggesting that within IP specifically, the accuracy and over-reliance clusters of that general taxonomy may be judged, by this elicitation method at least, as disproportionately salient relative to domains such as explainability or cybersecurity that are equally well represented in the general LLM-safety literature but were consistently deprioritised in our data (Section 3.3). Several of the inductively derived domains also correspond broadly to principles of consensus frameworks for trustworthy medical AI, such as fairness and universality (D3), robustness (D1 and D4), traceability (D4 and D6), usability (D8), and explainability (D6) in the FUTURE-AI guideline [46], whereas automation bias (D2) and deskilling (D5), both human-factors risks, do not map directly onto any single principle. This partial correspondence lends some face validity to the taxonomy while confirming that it is an empirical, data-driven grouping rather than a formal framework.

### 4.7. A Candidate Role for Multimodel LLM Elicitation in Risk Horizon-Scanning

We propose that structured, multimodel LLM elicitation of the kind demonstrated here may have a modest but genuine role as a rapid, low-cost complement to—never a substitute for—formal expert consensus methods (e.g., Delphi panels, nominal group technique [47]) in the early, horizon-scanning phase of risk characterisation for an emerging technology in a procedural subspecialty. Its comparative advantages are speed, minimal cost, and immediate replicability of the elicitation protocol itself (the standardised prompt in Supplementary Table S1 can be re-administered by any reader, although its outputs may vary across runs and model versions); its outputs are, however, hypotheses about the current state of AI-safety discourse as encoded in model training data, not validated evidence about real-world risk incidence, and must be treated accordingly. We see the most defensible next step as using the domains and constructs identified here (Table 3, Figure 1) as a structured starting point for a formal expert Delphi or nominal-group consensus exercise among IP specialists—which could also refine the present pragmatic structure into a more granular, layered taxonomy, for example by separating conceptually distinct aspects currently grouped within a single domain, and compare it with alternative integration strategies, such as independent multi-coder consensus or computational clustering of the risk statements—and, in parallel, for prospective, outcome-based empirical research of the kind that has begun to emerge for AI-assisted colonoscopy [32].

### 4.8. Training Data as a Determinant of the Elicited Risk Landscape

The material on which the models were trained is likely to be a major determinant of the results. General-purpose LLMs generate text by reproducing patterns learned from very large training corpora, and their outputs are shaped by the content, recency, and biases of those data [38,48]; the exact composition of these corpora is not publicly documented in detail for the systems studied. The risk landscape elicited here should therefore be read as a reflection of how AI-related risks are represented in the scientific, regulatory, and non-scientific material available to the models, up to their training cut-off and possibly through web content retrieved at the time of querying (Section 2.3), rather than as an independent appraisal of real-world risk. Two consequences follow. First, the salience of a risk in the outputs may track the volume and prominence of discourse about it rather than its clinical incidence: because IP-specific literature on AI safety remains limited, the models are likely to have extrapolated from better-documented areas, such as the general literature on AI safety in healthcare and on AI-assisted endoscopy [23,32], and biased or outdated content embedded in training data can be reproduced in model outputs [38]. Second, the training corpora of different developers are likely to overlap substantially, and the broad-data training paradigm of foundation models incentivises homogenisation, with defects potentially shared by the systems built upon them [49]; the five systems therefore cannot be regarded as fully independent informants, and the convergence observed across models may reflect common underlying literature and public discourse rather than independent confirmation of any given risk domain.

Conversely, several potentially relevant risk areas did not emerge as distinct domains in the model outputs, although some may be partly subsumed within broader domains (for example, liability within accountability in D6, or access within health equity in D3). These include medico-legal liability [50] and informed consent for AI-assisted procedures; regulatory obligations, such as conformity assessment and post-market monitoring, under frameworks such as the EU AI Act [33]; the economic consequences of costly AI-enabled platforms, including procurement, reimbursement, and dependence on proprietary vendor ecosystems; the environmental footprint of training and operating large AI models [49]; effects on patient–clinician communication and shared decision-making; and risks arising from patients’ own use of general-purpose chatbots before or after procedures [21,22]. Their absence should not be interpreted as evidence of lower importance; rather, it illustrates that an unconstrained, ten-item elicitation tends to favour the most prominent themes in the underlying discourse, and these areas warrant explicit consideration in future expert-led work.

### 4.9. Risks and Benefits as Two Faces of the Same Attributes

Several of the risk domains identified here correspond to attributes that are also widely presented as benefits of AI. Health equity is the clearest example: AI could extend expert-level image interpretation, navigation support, and decision support to lower-volume or resource-limited settings, thereby reducing disparities, yet the same systems may widen inequities if they are developed on unrepresentative data or deployed only where costly platforms are affordable [24,40]. Similarly, AI-based feedback improved novices’ bronchoscopy performance in a randomised trial conducted in a simulated setting [51], whereas sustained reliance on AI assistance may erode unassisted skills [27,32]; decision support intended to assist clinicians can generate alert fatigue [45]; and the aggregation of procedural data that may improve model performance also raises privacy and cybersecurity concerns [44]. Whether a given attribute behaves as a risk or a benefit therefore depends on how AI is designed, validated, implemented, and governed, rather than on the technology itself. Because our prompt requested risks only, it framed the outputs towards harms; the resulting taxonomy should not be read as a net benefit–risk assessment, and a balanced elicitation of paired benefits and risks for specific IP applications would be a natural extension of this work.

### 4.10. Limitations

Several limitations should be considered when interpreting these findings. First, the design used a single prompt formulation, administered once per model for full structured retention; prompt wording, temperature/sampling settings, and conversational framing are all known to influence LLM output, and different phrasings might elicit a different risk landscape. Second, although the protocol included an informal repeat administration per model, only one full structured transcript per model was retained for analysis, so output stability was not formally assessed and any impression of between-run consistency rests solely on an unquantified operator observation rather than on an independently archived and re-analysed second dataset; third, five models were selected for developer and platform diversity, not as an exhaustive or randomly sampled set of available LLMs, and both the specific models and their outputs will continue to evolve, so the exact risk landscape reported here should not be assumed to be reproducible with later model versions or even the same versions queried at a later date. Fourth, qualitative coding into constructs and domains was performed by a single coder—a generative AI system operating under direct human supervision and final approval by the corresponding author—and was only subsequently subjected to independent human validation by two reviewers; reliance on an AI coder for the initial classification, even when independently validated, is a material limitation for a qualitative classification task of this kind, compounded by a risk of circularity because the same model (Claude Sonnet 5) also generated one of the five analysed outputs; the complete item-level coding matrix is therefore provided (Supplementary Table S2) to allow independent re-coding by other investigators. Fifth, the risk statements analysed are LLM-generated text reflecting patterns in public training data and discourse about AI safety, not empirically validated clinical incidence data, expert consensus, or IP-specific ground truth; no claim is made that any individual risk statement, mechanism, or mitigation strategy is clinically accurate, complete, or ready for direct implementation. Sixth, the comparison with our prior human-expert survey is narrative and non-statistical, reflects two studies with different designs, instruments, and populations, and should not be read as a formal concordance or agreement analysis. Seventh, web search and other external tools were not specifically disabled, so a contribution of real-time web retrieval to individual outputs cannot be excluded. Eighth, the five systems cannot be considered fully independent informants, because their training corpora probably overlap; cross-model convergence therefore does not constitute independent confirmation of a risk domain. Ninth, impact and likelihood were self-assigned on non-anchored ordinal scales and are not calibrated measures of severity or probability. Tenth, the prompt requested risks only and may therefore have framed the outputs towards harms, without capturing the benefits associated with the same attributes. Taken together, these limitations mean the present findings should be read as a structured and transparent map of currently prominent AI-safety discourse concerning IP as reflected in five general-purpose LLMs—valuable, we suggest, for hypothesis generation and for scoping future expert-validated research, training curricula, and governance discussions, but not as a validated risk assessment or clinical guidance document in its own right.

## 5. Conclusions

Five general-purpose LLMs, queried in separate sessions with a single standardised prompt, enumerated and ranked a broadly convergent set of AI-related risks for IP, dominated by diagnostic/navigational accuracy and automation bias, with deskilling, generalisability/equity, and explainability/accountability listed by every model but consistently ranked lower. Contrasted descriptively with our own prior international survey of practising IP specialists, in which deskilling was the single highest-rated research priority, this lower ranking of deskilling by the LLMs is a key finding: LLM-generated rankings and human expert judgement in this domain may converge on which risks exist while diverging on their relative priority, which underscores the need for domain-expert input when AI-related risks are prioritised—an observation we offer as hypothesis-generating rather than conclusive. We propose structured multimodel LLM elicitation as a rapid, transparent, low-cost complement to, not a replacement for, formal expert consensus and prospective empirical research, and we offer the taxonomy developed here (Table 3, Figure 1) as a concrete, citable starting point for both.

## Figures and Tables

**Figure 1:**
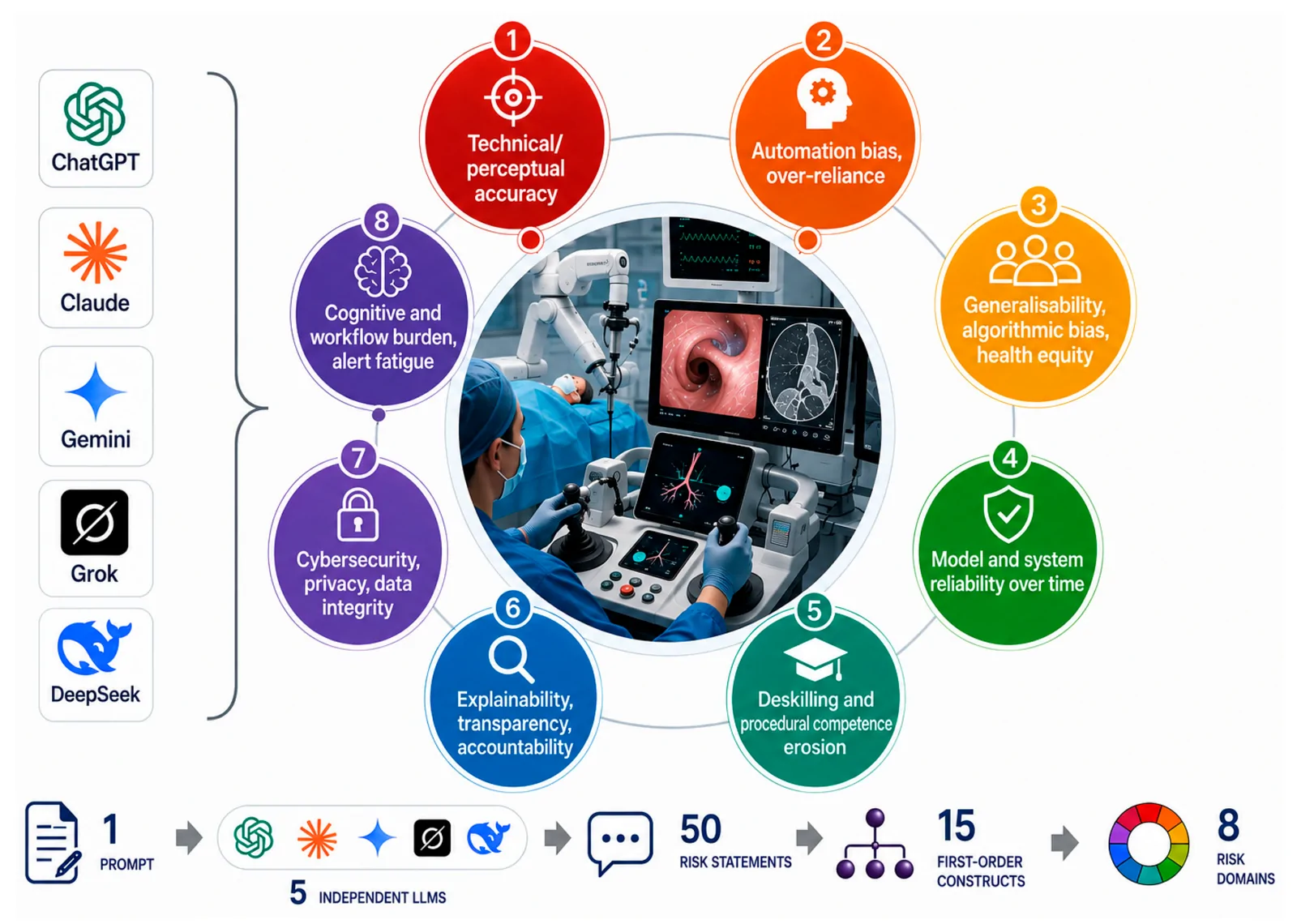
Eight-domain taxonomy of AI-related risks in interventional pulmonology. Five general-purpose LLMs, queried in separate sessions, generated 50 risk statements, which were inductively synthesised into 15 first-order constructs and eight higher-order risk domains. The taxonomy highlights the convergence and breadth of AI-related risk framing identified across the five models.

**Table 1:** Overview of the five AI systems queried and administration conditions.

Model	Developer	Mode	Platform	Session Type	Query Date
GPT-5.6 Luna	OpenAI	“Think”/reasoning mode	ChatGPT web app (Chrome)	New temporary chat; no history	21 August 2026
Claude Sonnet 5	Anthropic	Extended thinking (“reasoning”), Max effort	Claude.ai web app (Chrome)	New temporary chat; no history	21 August 2026
Gemini 3.1 Pro	Google	“Think”/extended reasoning	Gemini web app (Chrome)	New temporary chat; no history	21 August 2026
Grok 4.5	xAI	Structured analytical reasoning	Grok Web app (Chrome)	New temporary chat; no history	21 August 2026
DeepSeek-R1	DeepSeek	“Think”/deep reasoning mode	DeepSeek Web app (Chrome)	New temporary chat; no history	21 August 2026

**Table 2:** Per-model descriptive statistics and internal rank consistency. Impact and likelihood are ordinal, non-anchored ratings; means (SD) are shown as descriptive summaries only, and *p*-values are not reported (Section 2.6).

Model	Mean Impact (SD)	Mean Likelihood (SD)	Mean Impact × Likelihood	Spearman ρ (Rank vs. Impact × Likelihood)
ChatGPT	4.40 (0.70)	3.50 (0.53)	15.4	−0.93
Claude	3.50 (0.71)	3.30 (0.48)	11.4	−0.87
Gemini	3.90 (0.74)	3.70 (0.95)	14.2	−0.86
Grok	3.90 (0.88)	3.60 (0.84)	13.9	−0.89
DeepSeek	3.90 (0.74)	3.60 (0.52)	14.0	−0.88
All models (*N* = 50)	3.92 (0.78)	3.54 (0.68)	—	—

**Table 3:** Domain-level qualitative coding summary (*N* = 50 risk statements). Mean impact and likelihood values are descriptive summaries of ordinal, non-anchored ratings (Section 2.6).

Domain	Definition (Abbreviated)	Items (*n*)	Models (*n*/5)	Mean Impact	Mean Likelihood
D1	AI technical/perceptual accuracy (diagnostic, detection & navigational)	9	5/5	4.33	3.44
D2	Automation bias & over-reliance	6	5/5	4.17	4.00
D3	Generalisability, algorithmic bias & health equity	8	5/5	4.00	3.62
D4	Model & system reliability over time	5	4/5 (no DeepSeek)	4.20	3.00
D5	Deskilling & erosion of procedural competence	5	5/5	3.80	3.80
D6	Explainability, transparency & accountability	9	5/5	3.33	3.67
D7	Cybersecurity, privacy & data integrity	4	4/5 (no Claude)	4.50	2.75
D8	Cognitive & workflow burden (alert fatigue)	4	4/5 (no Claude)	3.00	3.75

## Data Availability

The complete dataset analysed in this study (the verbatim standardised prompt, the full text of all 50 AI-generated risk statements, and the qualitative coding matrix) is provided in the Supplementary Materials (Tables S1 and S2). The Python analysis code and full console output are provided as Supplementary File S1. No restrictions apply to the availability of these materials.

## Supplementary Materials

The following supporting information can be downloaded at: https://www.mdpi.com/article/10.3390/jcm15197340/s1, Table S1: Verbatim standardised prompt administered to all five AI systems. Table S2: Full coded matrix of all 50 risk statements (model, rank, verbatim title, impact, likelihood, domain code, construct code). Figures S1–S5: Session screenshots documenting the query conditions for ChatGPT, Claude, Gemini, Grok, and DeepSeek, respectively. File S1: Python analysis script and full console output underlying Table 2 and Table 3 and Section 3.

## Abbreviations

The following abbreviations are used in this manuscript:

AI	Artificial intelligence
GenAI	Generative artificial intelligence
IP	Interventional pulmonology
LLM	Large language model
EBUS	Endobronchial ultrasound
SD	Standard deviation
