# Supplementary material for: Artificial Intelligence-Related Risks in Interventional Pulmonology: An Exploratory Enumeration and Ranking Study Across Five General-Purpose Large Language Models
Source: J Clin Med. 2026 Sep 22;15(19):7340. doi: 10.3390/jcm15197340 (PMC13665493; doi:10.3390/jcm15197340)
Supplement: Supplementary file 1 [file jcm-15-07340-s001.zip › jcm-4570926-supplementary.pdf]

Supplementary Materials

Contents:

- ***Table S1*** *(verbatim standardised prompt);*
- ***Table S2*** *(full coded matrix of all 50 risk statements);*
- ***Figures S1–S5*** *(session screenshots);*
- ***File S1*** *(Python analysis script and console output).*

Table S1. Verbatim standardised prompt administered to all five AI systems

```
You are participating in a structured exercise to help identify potential risks associated with the use of artificial intelligence (AI) in interventional pulmonology.

Interventional pulmonology includes procedures and clinical activities such as diagnostic and therapeutic bronchoscopy, endobronchial ultrasound, navigation bronchoscopy, pleural procedures, tissue acquisition, procedural planning, image interpretation, clinical decision-making, documentation, workflow management and training.

Consider current and foreseeable applications of AI in this field, including but not limited to image analysis, procedural planning, navigation, lesion characterisation, decision support, workflow optimisation, education and automation.

Your task is to identify potential risks, harms, limitations or unintended consequences that could arise from the use of AI in interventional pulmonology. Do not assume that AI is inherently beneficial, harmful or safe. Consider both established and emerging risks, across clinical, technical, human factors, ethical, professional, organisational and societal dimensions.

Please identify exactly 10 distinct potential risks.

For each risk, provide:

1\. A concise risk title (maximum 10 words).

2\. A brief description (1-2 sentences).

3\. The mechanism through which AI could produce or contribute to this risk.

4\. The principal affected stakeholders (e.g. patient, physician/proceduralist, healthcare system, researchers, society).

5\. A qualitative impact score from 1 (minimal) to 5 (potentially catastrophic).

6\. A qualitative likelihood score from 1 (very unlikely) to 5 (very likely).

7\. One practical mitigation strategy.

After listing the 10 risks, rank them from highest to lowest overall priority, and briefly justify your top-ranked risk.

Avoid repeating conceptually overlapping risks. Do not cite specific publications unless explicitly requested. Do not provide general recommendations unrelated to the identified risks. The purpose of this exercise is not to test factual or
```

```
medical knowledge, but to map the risk landscape associated with AI in interventional pulmonology as you conceptualise it.

Present your answer as a structured table with the following columns:

Rank \| Risk title \| Description \| Mechanism \| Affected stakeholder(s) \| Impact (1-5) \| Likelihood (1-5) \| Mitigation strategy
```

Table S2. Full coded matrix of all 50 elicited risk statements

Model, rank (as stated by the model), verbatim risk title, self-assigned impact (1–5) and likelihood (1–5) scores, and primary domain/construct code assigned during qualitative coding (Section 2.5 of the main text; full domain and construct definitions in Table 2 of the main text and Section 1 above). Where a statement's title spanned two constructs, both codes are shown separated by "/", with the first being the primary code used in all quantitative counts.

#	Model	Rank	Risk title (verbatim)	Impact	Likelihood	Domain	Construct
1	ChatGPT	1	AI-driven diagnostic or navigational error	5	4	D1	c1a/c1b
2	ChatGPT	2	Automation bias and over-reliance	5	4	D2	c2a
3	ChatGPT	3	Poor generalisation across settings	5	4	D3	c3a
4	ChatGPT	4	Hidden model drift over time	5	3	D4	c4a
5	ChatGPT	5	Automation-induced workflow and procedural failures	4	4	D4	c4b
6	ChatGPT	6	Cybersecurity and data compromise	5	3	D7	c7a
7	ChatGPT	7	Unequal performance and access	4	3	D3	c3c/c3b
8	ChatGPT	8	Deskilling and loss of procedural expertise	4	3	D5	c5a
9	ChatGPT	9	Opacity and unclear accountability	4	3	D6	c6a/c6b
10	ChatGPT	10	Alert fatigue and information overload	3	4	D8	c8a
11	Claude	1	Misclassification of lesion malignancy by AI	5	3	D1	c1a
12	Claude	2	Automation bias during AI-guided navigation	4	3	D2	c2a
13	Claude	3	Algorithmic bias across patient subgroups	4	3	D3	c3b
14	Claude	4	Undetected AI performance drift after deployment	4	3	D4	c4a
15	Claude	5	Deskilling of proceduralists through AI dependence	3	4	D5	c5a
16	Claude	6	Opacity of AI outputs limiting clinical scrutiny	3	4	D6	c6a
17	Claude	7	Unequal access to AI-enabled procedural technology	3	4	D3	c3c
18	Claude	8	False reassurance from AI tissue-adequacy assessment	3	3	D1	c1a
19	Claude	9	Overdiagnosis from AI-enhanced detection sensitivity	3	3	D1	c1c
20	Claude	10	Ambiguous liability for AI-related adverse events	3	3	D6	c6b
21	Gemini	1	CT-to-Body Divergence Navigation Failures	5	4	D1	c1b
22	Gemini	2	Over-trust in Algorithmic Outputs	4	5	D2	c2a
23	Gemini	3	Erosion of Core Procedural Competencies	4	4	D5	c5a
24	Gemini	4	AI Generalizability Failure Across Populations	4	4	D3	c3a
25	Gemini	5	Systemic Vulnerability to IT Failures	5	2	D4	c4b
26	Gemini	6	Lack of Clinical Explainability	3	5	D6	c6a
27	Gemini	7	Unclear Legal Accountability for Errors	4	3	D6	c6b
28	Gemini	8	Compromise of Identifiable Medical Data	4	3	D7	c7a
29	Gemini	9	Intra-procedural Alert Fatigue	3	4	D8	c8a
30	Gemini	10	AI-Driven Triage and Resource Misallocation	3	3	D2	c2a
31	Grok	1	Incorrect AI lesion characterisation	5	4	D1	c1a
32	Grok	2	Procedural navigation guidance failure	5	3	D1	c1b
33	Grok	3	Automation bias and over-reliance	4	5	D2	c2a
34	Grok	4	Deskilling of core technical competencies	4	4	D5	c5a
35	Grok	5	Algorithmic bias across patient subgroups	4	4	D3	c3b
36	Grok	6	Black-box opacity impairing informed consent and accountability	3	4	D6	c6a/c6b
37	Grok	7	Cybersecurity and data-integrity compromise	5	2	D7	c7a

38	Grok	8	Workflow friction and cognitive overload	3	4	D8	c8a
39	Grok	9	Liability diffusion and defensive practice distortion	3	3	D6	c6b
40	Grok	10	Propagation of outdated or institution-specific practice patterns	3	3	D4	c4a
41	DeepSeek	1	Automation bias in AI lesion characterisation causing missed malignancy	5	4	D2	c2a
42	DeepSeek	2	AI navigation or robotic actuation failure causing airway/vascular injury	5	3	D1	c1b
43	DeepSeek	3	Algorithmic underperformance in rare diseases or underrepresented populations	4	4	D3	c3a/c3b
44	DeepSeek	4	Procedural deskilling and loss of bronchoscopic competence	4	4	D5	c5a
45	DeepSeek	5	Liability and accountability gaps after AI-related adverse events	4	4	D6	c6b
46	DeepSeek	6	AI false positives and overdiagnosis leading to unnecessary invasive biopsies	3	4	D1	c1c
47	DeepSeek	7	Privacy breach or misuse of procedural imaging and patient data	4	3	D7	c7a
48	DeepSeek	8	Generative AI documentation errors altering clinical records	3	4	D6	c6c
49	DeepSeek	9	Unequal access and infrastructure gaps exacerbating health disparities	4	3	D3	c3c
50	DeepSeek	10	Real-time alert fatigue and cognitive overload during bronchoscopy	3	3	D8	c8a

Figures S1–S5. Session screenshots

Screenshots of the query session for each of the five AI systems, documenting the temporary/incognito session state, model/version selector, and query interface at the time of data collection (21 August 2026). These are provided for transparency and to substantiate the session conditions reported in Table 1 of the main text; they are not a data source for the quantitative analysis, which used the structured table output only (Section 2.4 of the main text).

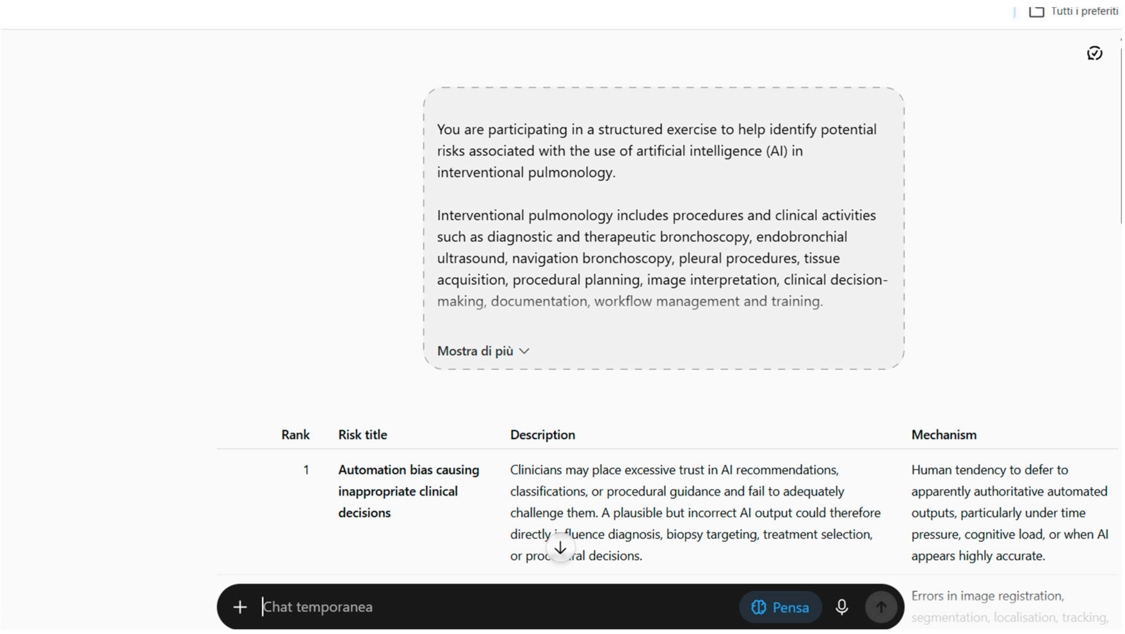

Figure S1. Session screenshot — ChatGPT (GPT-5.6 Luna), 21 August 2026.

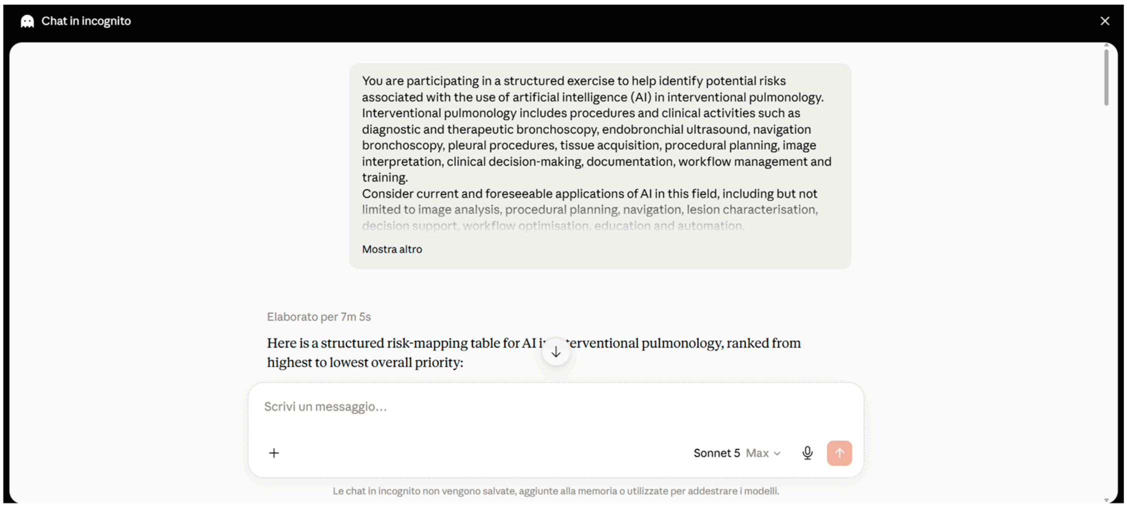

Figure S2. Session screenshot — Claude (Claude Sonnet 5), 21 August 2026.

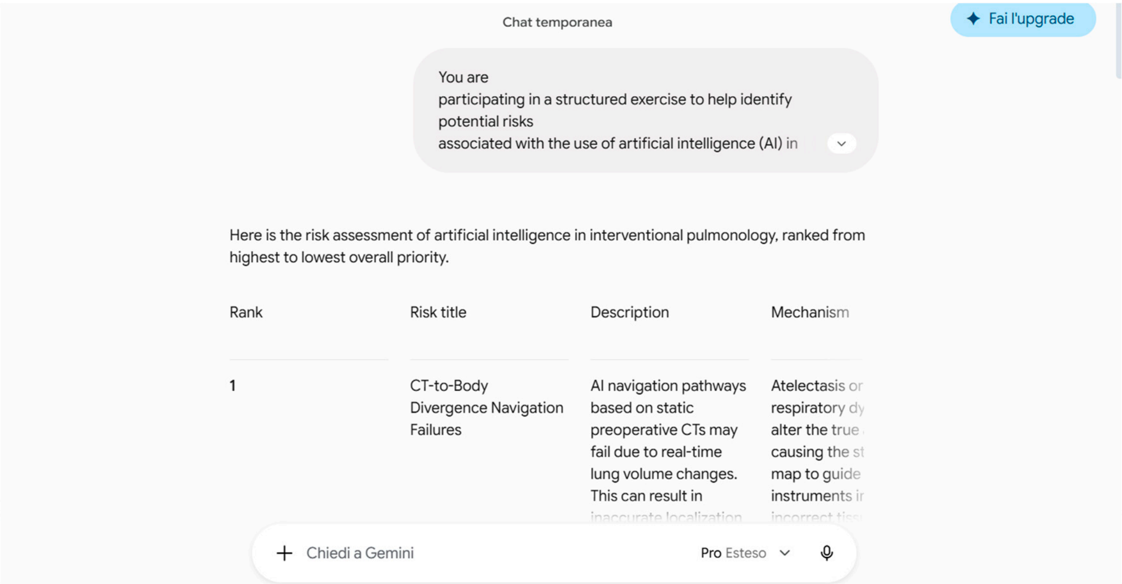

Figure S3. Session screenshot — Gemini (Gemini 3.1 Pro), 21 August 2026.

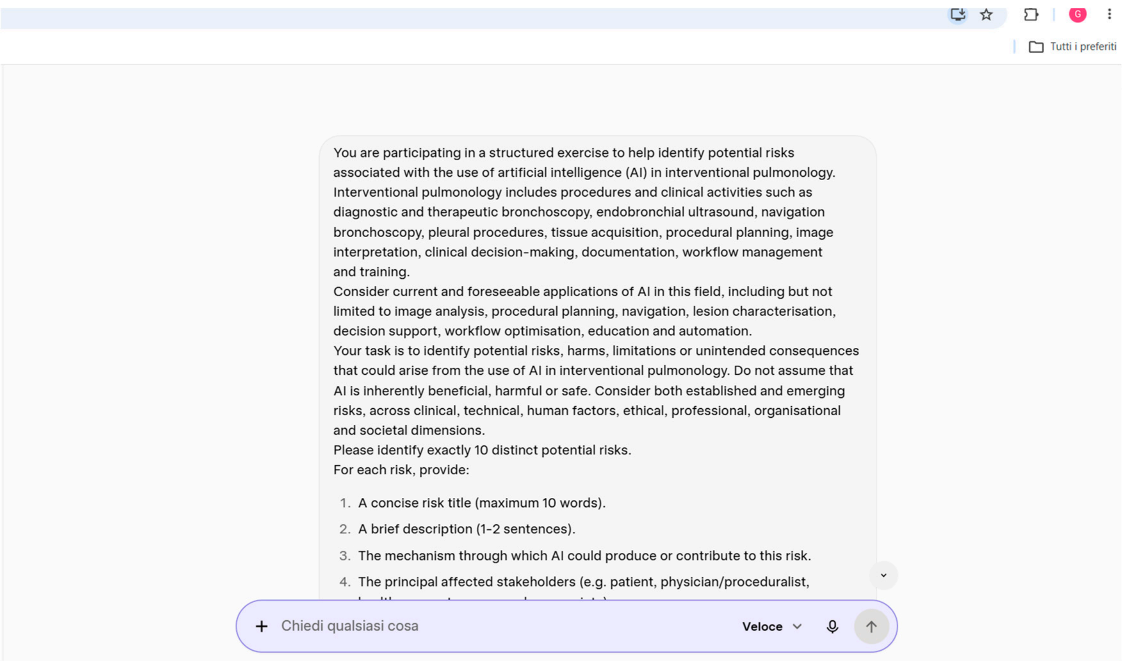

Figure S4. Session screenshot — Grok (Grok 4.5), 21 August 2026.

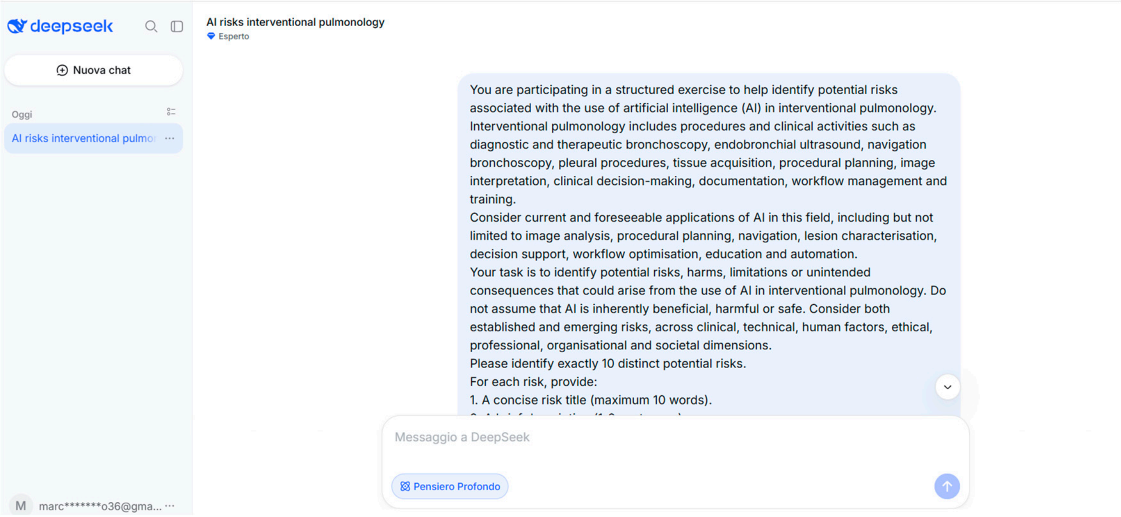

Figure S5. Session screenshot — DeepSeek (DeepSeek-R1), 21 August 2026.

File S1. Python analysis script and console output

The complete Python script used to compute all descriptive and exploratory statistics reported in Tables 2 and 3 and Section 3 of the main text, and its full, unedited console output, are reproduced below for transparency and reproducibility. The script operates on the dataset in Table S2 above (hard-coded within the script from the verbatim source data; see Section 2.4 of the main text).

S1.1. Analysis script (coding_analysis.py)

```
#!/usr/bin/env python3
# -*- coding: utf-8 -*-
"""
Qualitative coding matrix + descriptive statistics for the 50 risk statements
elicited from 5 general-purpose LLMs (ChatGPT, Claude, Gemini, Grok, DeepSeek),
10 ranked risks each, from outputs_raw.md (verbatim transcription).

All titles, impact and likelihood scores below are transcribed verbatim from
/home/claude/manuscript/outputs_raw.md (the OUTPUTS.docx the user supplied).
No values are invented. Domain/construct codes are the author's (Claude's)
qualitative coding, performed as a single coder (see Methods/Limitations).
"""
import json
import statistics as st
from scipy import stats as sps

# ----------------------------------------------------------------------
# RAW DATA: (model, rank, title, impact, likelihood, domain, construct, note)
# ----------------------------------------------------------------------
# Domain codes:
# D1 AI Technical/Perceptual Accuracy (Diagnostic Interpretation, Detection & Navigational
Targeting)
# D2 Automation Bias & Over-Reliance
# D3 Generalisability, Algorithmic Bias & Health Equity
# D4 Model & System Reliability Over Time
# D5 Deskilling & Erosion of Procedural Competence
# D6 Explainability, Transparency & Accountability
# D7 Cybersecurity, Privacy & Data Integrity
# D8 Cognitive & Workflow Burden (Alert Fatigue/Overload)
#
# Construct codes (sub-construct within domain):
# c1a diagnostic/interpretive misclassification (incl. false reassurance)
# c1b navigational/targeting/registration failure
# c1c overdiagnosis / excess sensitivity (false-positive direction)
# c2a automation bias / over-reliance / reduced vigilance
# c3a generalisability / external validity across settings-populations
# c3b algorithmic/demographic bias & subgroup underperformance
# c3c unequal access to AI-enabled care (resource/infrastructure disparity)
# c4a performance drift / obsolescence over time
# c4b system/workflow continuity & integration failure (incl. IT outages)
# c5a deskilling
# c6a opacity / black-box explainability
# c6b liability / accountability ambiguity
# c6c AI-generated documentation / record-integrity error
# c7a cybersecurity / data breach / privacy
# c8a alert fatigue / information & cognitive overload

DATA = [
 # ChatGPT
 ("ChatGPT", 1, "AI-driven diagnostic or navigational error", 5, 4, "D1", "c1a/c1b", "combined
item"),
 ("ChatGPT", 2, "Automation bias and over-reliance", 5, 4, "D2", "c2a", ""),
 ("ChatGPT", 3, "Poor generalisation across settings", 5, 4, "D3", "c3a", ""),
 ("ChatGPT", 4, "Hidden model drift over time", 5, 3, "D4", "c4a", ""),
 ("ChatGPT", 5, "Automation-induced workflow and procedural failures", 4, 4, "D4", "c4b", ""),
 ("ChatGPT", 6, "Cybersecurity and data compromise", 5, 3, "D7", "c7a", ""),
 ("ChatGPT", 7, "Unequal performance and access", 4, 3, "D3", "c3c/c3b", "combined item"),
 ("ChatGPT", 8, "Deskilling and loss of procedural expertise", 4, 3, "D5", "c5a", ""),
 ("ChatGPT", 9, "Opacity and unclear accountability", 4, 3, "D6", "c6a/c6b", "combined item"),
 ("ChatGPT", 10, "Alert fatigue and information overload", 3, 4, "D8", "c8a", ""),
 # Claude
 ("Claude", 1, "Misclassification of lesion malignancy by AI", 5, 3, "D1", "c1a", ""),
 ("Claude", 2, "Automation bias during AI-guided navigation", 4, 3, "D2", "c2a", "navigation
context"),
 ("Claude", 3, "Algorithmic bias across patient subgroups", 4, 3, "D3", "c3b", ""),
 ("Claude", 4, "Undetected AI performance drift after deployment", 4, 3, "D4", "c4a", ""),
 ("Claude", 5, "Deskilling of proceduralists through AI dependence", 3, 4, "D5", "c5a", ""),
 ("Claude", 6, "Opacity of AI outputs limiting clinical scrutiny", 3, 4, "D6", "c6a", ""),
 ("Claude", 7, "Unequal access to AI-enabled procedural technology", 3, 4, "D3", "c3c", ""),
```

```
 ("Claude", 8, "False reassurance from AI tissue-adequacy assessment", 3, 3, "D1", "c1a", "adequacy-
specific"),
 ("Claude", 9, "Overdiagnosis from AI-enhanced detection sensitivity", 3, 3, "D1", "c1c", ""),
 ("Claude", 10, "Ambiguous liability for AI-related adverse events", 3, 3, "D6", "c6b", ""),
 # Gemini
 ("Gemini", 1, "CT-to-Body Divergence Navigation Failures", 5, 4, "D1", "c1b", ""),
 ("Gemini", 2, "Over-trust in Algorithmic Outputs", 4, 5, "D2", "c2a", ""),
 ("Gemini", 3, "Erosion of Core Procedural Competencies", 4, 4, "D5", "c5a", ""),
 ("Gemini", 4, "AI Generalizability Failure Across Populations", 4, 4, "D3", "c3a", ""),
 ("Gemini", 5, "Systemic Vulnerability to IT Failures", 5, 2, "D4", "c4b", ""),
 ("Gemini", 6, "Lack of Clinical Explainability", 3, 5, "D6", "c6a", ""),
 ("Gemini", 7, "Unclear Legal Accountability for Errors", 4, 3, "D6", "c6b", ""),
 ("Gemini", 8, "Compromise of Identifiable Medical Data", 4, 3, "D7", "c7a", ""),
 ("Gemini", 9, "Intra-procedural Alert Fatigue", 3, 4, "D8", "c8a", ""),
 ("Gemini", 10, "AI-Driven Triage and Resource Misallocation", 3, 3, "D2", "c2a", "triage/resource
variant"),
 # Grok
 ("Grok", 1, "Incorrect AI lesion characterisation", 5, 4, "D1", "c1a", ""),
 ("Grok", 2, "Procedural navigation guidance failure", 5, 3, "D1", "c1b", ""),
 ("Grok", 3, "Automation bias and over-reliance", 4, 5, "D2", "c2a", ""),
 ("Grok", 4, "Deskilling of core technical competencies", 4, 4, "D5", "c5a", ""),
 ("Grok", 5, "Algorithmic bias across patient subgroups", 4, 4, "D3", "c3b", ""),
 ("Grok", 6, "Black-box opacity impairing informed consent and accountability", 3, 4, "D6",
"c6a/c6b", "combined item"),
 ("Grok", 7, "Cybersecurity and data-integrity compromise", 5, 2, "D7", "c7a", ""),
 ("Grok", 8, "Workflow friction and cognitive overload", 3, 4, "D8", "c8a", ""),
 ("Grok", 9, "Liability diffusion and defensive practice distortion", 3, 3, "D6", "c6b", ""),
 ("Grok", 10, "Propagation of outdated or institution-specific practice patterns", 3, 3, "D4",
"c4a", "obsolescence variant"),
 # DeepSeek
 ("DeepSeek", 1, "Automation bias in AI lesion characterisation causing missed malignancy", 5, 4,
"D2", "c2a", "diagnostic-outcome overlap w/ D1"),
 ("DeepSeek", 2, "AI navigation or robotic actuation failure causing airway/vascular injury", 5, 3,
"D1", "c1b", ""),
 ("DeepSeek", 3, "Algorithmic underperformance in rare diseases or underrepresented populations", 4,
4, "D3", "c3a/c3b", "combined item"),
 ("DeepSeek", 4, "Procedural deskilling and loss of bronchoscopic competence", 4, 4, "D5", "c5a",
""),
 ("DeepSeek", 5, "Liability and accountability gaps after AI-related adverse events", 4, 4, "D6",
"c6b", ""),
 ("DeepSeek", 6, "AI false positives and overdiagnosis leading to unnecessary invasive biopsies", 3,
4, "D1", "c1c", ""),
 ("DeepSeek", 7, "Privacy breach or misuse of procedural imaging and patient data", 4, 3, "D7",
"c7a", ""),
 ("DeepSeek", 8, "Generative AI documentation errors altering clinical records", 3, 4, "D6", "c6c",
""),
 ("DeepSeek", 9, "Unequal access and infrastructure gaps exacerbating health disparities", 4, 3,
"D3", "c3c", ""),
 ("DeepSeek", 10, "Real-time alert fatigue and cognitive overload during bronchoscopy", 3, 3, "D8",
"c8a", ""),
]

assert len(DATA) == 50, f"Expected 50 rows, got {len(DATA)}"
for m in ["ChatGPT","Claude","Gemini","Grok","DeepSeek"]:
    n = sum(1 for r in DATA if r[0]==m)
    assert n==10, f"{m} has {n} rows"

MODELS = ["ChatGPT","Claude","Gemini","Grok","DeepSeek"]
DOMAIN_NAMES = {
 "D1":"AI Technical/Perceptual Accuracy (Diagnostic Interpretation, Detection & Navigational
Targeting)",
 "D2":"Automation Bias & Over-Reliance",
 "D3":"Generalisability, Algorithmic Bias & Health Equity",
 "D4":"Model & System Reliability Over Time",
 "D5":"Deskilling & Erosion of Procedural Competence",
 "D6":"Explainability, Transparency & Accountability",
 "D7":"Cybersecurity, Privacy & Data Integrity",
 "D8":"Cognitive & Workflow Burden (Alert Fatigue/Overload)",
}

print("="*100)
print("1. GRAND DESCRIPTIVE STATISTICS (N=50 risk statements)")
print("="*100)
impacts = [r[3] for r in DATA]
likelis = [r[4] for r in DATA]
print(f"Impact:     mean={st.mean(impacts):.2f}  sd={st.stdev(impacts):.2f}
median={st.median(impacts)}  min={min(impacts)} max={max(impacts)}")
print(f"Likelihood: mean={st.mean(likelis):.2f}  sd={st.stdev(likelis):.2f}
median={st.median(likelis)}  min={min(likelis)} max={max(likelis)}")
rho, p = sps.pearsonr(impacts, likelis)
print(f"Pearson r (impact, likelihood) across all 50 items: r={rho:.3f}, p={p:.3f} (descriptive;
items are not independent - 10 per model)")
rho_s, p_s = sps.spearmanr(impacts, likelis)
print(f"Spearman rho (impact, likelihood): rho={rho_s:.3f}, p={p_s:.3f}")
```

```
print()
print("="*100)
print("2. PER-MODEL DESCRIPTIVE STATISTICS")
print("="*100)
per_model_stats = {}
for m in MODELS:
    rows = [r for r in DATA if r[0]==m]
    im = [r[3] for r in rows]; li = [r[4] for r in rows]
    ranks = [r[1] for r in rows]
    prod = [a*b for a,b in zip(im,li)]
    rho_rank_prod, p_rank_prod = sps.spearmanr(ranks, prod)  # expect negative (higher rank number =
lower priority)
    per_model_stats[m] = dict(
        mean_impact=st.mean(im), sd_impact=st.stdev(im),
        mean_likeli=st.mean(li), sd_likeli=st.stdev(li),
        mean_product=st.mean(prod),
        rho_rank_vs_product=rho_rank_prod, p_rank_vs_product=p_rank_prod,
    )
    print(f"{m:10s}  impact {st.mean(im):.2f}±{st.stdev(im):.2f}   likelihood
{st.mean(li):.2f}±{st.stdev(li):.2f}   "
          f"impact*likelihood mean={st.mean(prod):.2f}   Spearman(rank, impact*likelihood)
rho={rho_rank_prod:.3f} (p={p_rank_prod:.3f})")

print()
print("="*100)
print("3. DOMAIN-LEVEL CONVERGENCE (primary-coded domain; first code before '/')")
print("="*100)
def primary_domain(row):
    return row[5]
def primary_construct(row):
    c = row[6]
    return c.split("/")[0]

domain_items = {d: [] for d in DOMAIN_NAMES}
for r in DATA:
    d = primary_domain(r)
    domain_items[d].append(r)

domain_summary = []
for d, name in DOMAIN_NAMES.items():
    items = domain_items[d]
    models_present = sorted(set(r[0] for r in items), key=MODELS.index)
    models_absent = [m for m in MODELS if m not in models_present]
    im = [r[3] for r in items]; li=[r[4] for r in items]
    ranks_by_model = {}
    for r in items:
        ranks_by_model.setdefault(r[0], []).append(r[1])
    best_rank_per_model = {m: min(v) for m,v in ranks_by_model.items()}
    domain_summary.append(dict(
        domain=d, name=name, n_items=len(items), n_models=len(models_present),
        models_present=models_present, models_absent=models_absent,
        mean_impact=st.mean(im), mean_likeli=st.mean(li),
        best_rank_per_model=best_rank_per_model,
    ))
    print(f"\n{d} — {name}")
    print(f"  items={len(items)}  models represented={len(models_present)}/5 ({',
'.join(models_present)})")
    if models_absent:
        print(f"  NOT represented (as primary code) in: {', '.join(models_absent)}")
    print(f"  mean impact={st.mean(im):.2f}  mean likelihood={st.mean(li):.2f}")
    print(f"  best (highest-priority / lowest-numbered) rank contributed per model:
{best_rank_per_model}")

print()
print("="*100)
print("4. TOP-2 (RANK 1+2) DOMAIN CONCENTRATION ACROSS MODELS")
print("="*100)
top2 = [r for r in DATA if r[1] in (1,2)]
assert len(top2)==10
from collections import Counter
top2_domain_counts = Counter(primary_domain(r) for r in top2)
for d,c in top2_domain_counts.most_common():
    print(f"  {d} ({DOMAIN_NAMES[d]}): {c}/10 of all rank-1/rank-2 items")
top2_construct_counts = Counter(primary_construct(r) for r in top2)
print("  by construct:", dict(top2_construct_counts))

rank1 = [r for r in DATA if r[1]==1]
rank1_domain_counts = Counter(primary_domain(r) for r in rank1)
print(f"\n  Rank-1 only, by domain: {dict(rank1_domain_counts)}")
rank2 = [r for r in DATA if r[1]==2]
rank2_domain_counts = Counter(primary_domain(r) for r in rank2)
```

```
print(f"  Rank-2 only, by domain: {dict(rank2_domain_counts)}")

print()
print("="*100)
print("5. CONSTRUCT-LEVEL FULL TABLE (for supplementary / methods cross-check)")
print("="*100)
construct_items = {}
for r in DATA:
    c = primary_construct(r)
    construct_items.setdefault(c, []).append(r)
for c in sorted(construct_items):
    items = construct_items[c]
    models_present = sorted(set(r[0] for r in items), key=MODELS.index)
    print(f"  {c}: n={len(items)}  models={len(models_present)}/5 ({', '.join(models_present)})")

print()
print("="*100)
print("6. FULL DOMAIN x MODEL MATRIX (counts)")
print("="*100)
header = "Domain".ljust(4) + "".join(m.ljust(11) for m in MODELS) + "Total  Models"
print(header)
for d in DOMAIN_NAMES:
    row_counts = []
    for m in MODELS:
        n = sum(1 for r in DATA if r[0]==m and primary_domain(r)==d)
        row_counts.append(n)
    total_items = sum(row_counts)
    n_models = sum(1 for c in row_counts if c>0)
    print(d.ljust(4) + "".join(str(c).ljust(11) for c in row_counts) + f"{total_items}
{n_models}/5")

# Export structured JSON for downstream doc-building scripts
export = {
  "data": DATA,
  "domain_names": DOMAIN_NAMES,
  "grand": {"mean_impact": st.mean(impacts), "sd_impact": st.stdev(impacts),
            "mean_likelihood": st.mean(likelis), "sd_likelihood": st.stdev(likelis),
            "pearson_r": rho, "pearson_p": p},
  "per_model": per_model_stats,
  "domain_summary": domain_summary,
  "top2_domain_counts": dict(top2_domain_counts),
  "rank1_domain_counts": dict(rank1_domain_counts),
  "rank2_domain_counts": dict(rank2_domain_counts),
}
with open("/home/claude/manuscript/analysis_export.json","w") as f:
    json.dump(export, f, indent=2, default=str)
print("\nExported -> analysis_export.json")
```

S1.2. Console output

```
==========================================================================================
1. GRAND DESCRIPTIVE STATISTICS (N=50 risk statements)
==========================================================================================
Impact:     mean=3.92  sd=0.78  median=4.0  min=3 max=5
Likelihood: mean=3.54  sd=0.68  median=4.0  min=2 max=5
Pearson r (impact, likelihood) across all 50 items: r=-0.188, p=0.192 (descriptive; items are not
independent - 10 per model)
Spearman rho (impact, likelihood): rho=-0.155, p=0.281


==========================================================================================
2. PER-MODEL DESCRIPTIVE STATISTICS
==========================================================================================
ChatGPT     impact 4.40±0.70   likelihood 3.50±0.53   impact*likelihood mean=15.40   Spearman(rank,
impact*likelihood) rho=-0.934 (p=0.000)
Claude      impact 3.50±0.71   likelihood 3.30±0.48   impact*likelihood mean=11.40   Spearman(rank,
impact*likelihood) rho=-0.874 (p=0.001)
Gemini      impact 3.90±0.74   likelihood 3.70±0.95   impact*likelihood mean=14.20   Spearman(rank,
impact*likelihood) rho=-0.858 (p=0.001)
Grok        impact 3.90±0.88   likelihood 3.60±0.84   impact*likelihood mean=13.90   Spearman(rank,
impact*likelihood) rho=-0.890 (p=0.001)
DeepSeek    impact 3.90±0.74   likelihood 3.60±0.52   impact*likelihood mean=14.00   Spearman(rank,
impact*likelihood) rho=-0.881 (p=0.001)


==========================================================================================
3. DOMAIN-LEVEL CONVERGENCE (primary-coded domain; first code before '/')
==========================================================================================


D1 — AI Technical/Perceptual Accuracy (Diagnostic Interpretation, Detection & Navigational
Targeting)
  items=9  models represented=5/5 (ChatGPT, Claude, Gemini, Grok, DeepSeek)
  mean impact=4.33  mean likelihood=3.44
  best (highest-priority / lowest-numbered) rank contributed per model: {'ChatGPT': 1, 'Claude': 1,
'Gemini': 1, 'Grok': 1, 'DeepSeek': 2}


D2 — Automation Bias & Over-Reliance
  items=6  models represented=5/5 (ChatGPT, Claude, Gemini, Grok, DeepSeek)
  mean impact=4.17  mean likelihood=4.00
  best (highest-priority / lowest-numbered) rank contributed per model: {'ChatGPT': 2, 'Claude': 2,
'Gemini': 2, 'Grok': 3, 'DeepSeek': 1}


D3 — Generalisability, Algorithmic Bias & Health Equity
  items=8  models represented=5/5 (ChatGPT, Claude, Gemini, Grok, DeepSeek)
  mean impact=4.00  mean likelihood=3.62
  best (highest-priority / lowest-numbered) rank contributed per model: {'ChatGPT': 3, 'Claude': 3,
'Gemini': 4, 'Grok': 5, 'DeepSeek': 3}


D4 — Model & System Reliability Over Time
  items=5  models represented=4/5 (ChatGPT, Claude, Gemini, Grok)
  NOT represented (as primary code) in: DeepSeek
  mean impact=4.20  mean likelihood=3.00
  best (highest-priority / lowest-numbered) rank contributed per model: {'ChatGPT': 4, 'Claude': 4,
'Gemini': 5, 'Grok': 10}


D5 — Deskilling & Erosion of Procedural Competence
  items=5  models represented=5/5 (ChatGPT, Claude, Gemini, Grok, DeepSeek)
  mean impact=3.80  mean likelihood=3.80
  best (highest-priority / lowest-numbered) rank contributed per model: {'ChatGPT': 8, 'Claude': 5,
'Gemini': 3, 'Grok': 4, 'DeepSeek': 4}


D6 — Explainability, Transparency & Accountability
  items=9  models represented=5/5 (ChatGPT, Claude, Gemini, Grok, DeepSeek)
  mean impact=3.33  mean likelihood=3.67
  best (highest-priority / lowest-numbered) rank contributed per model: {'ChatGPT': 9, 'Claude': 6,
'Gemini': 6, 'Grok': 6, 'DeepSeek': 5}


D7 — Cybersecurity, Privacy & Data Integrity
  items=4  models represented=4/5 (ChatGPT, Gemini, Grok, DeepSeek)
  NOT represented (as primary code) in: Claude
  mean impact=4.50  mean likelihood=2.75
  best (highest-priority / lowest-numbered) rank contributed per model: {'ChatGPT': 6, 'Gemini': 8,
'Grok': 7, 'DeepSeek': 7}


D8 — Cognitive & Workflow Burden (Alert Fatigue/Overload)
  items=4  models represented=4/5 (ChatGPT, Gemini, Grok, DeepSeek)
  NOT represented (as primary code) in: Claude
  mean impact=3.00  mean likelihood=3.75
  best (highest-priority / lowest-numbered) rank contributed per model: {'ChatGPT': 10, 'Gemini': 9,
'Grok': 8, 'DeepSeek': 10}
```

```
================================================================================
4. TOP-2 (RANK 1+2) DOMAIN CONCENTRATION ACROSS MODELS
================================================================================
  D1 (AI Technical/Perceptual Accuracy (Diagnostic Interpretation, Detection & Navigational
Targeting)): 6/10 of all rank-1/rank-2 items
  D2 (Automation Bias & Over-Reliance): 4/10 of all rank-1/rank-2 items
  by construct: {'c1a': 3, 'c2a': 4, 'c1b': 3}

  Rank-1 only, by domain: {'D1': 4, 'D2': 1}
  Rank-2 only, by domain: {'D2': 3, 'D1': 2}

================================================================================
5. CONSTRUCT-LEVEL FULL TABLE (for supplementary / methods cross-check)
================================================================================
  c1a: n=4  models=3/5 (ChatGPT, Claude, Grok)
  c1b: n=3  models=3/5 (Gemini, Grok, DeepSeek)
  c1c: n=2  models=2/5 (Claude, DeepSeek)
  c2a: n=6  models=5/5 (ChatGPT, Claude, Gemini, Grok, DeepSeek)
  c3a: n=3  models=3/5 (ChatGPT, Gemini, DeepSeek)
  c3b: n=2  models=2/5 (Claude, Grok)
  c3c: n=3  models=3/5 (ChatGPT, Claude, DeepSeek)
  c4a: n=3  models=3/5 (ChatGPT, Claude, Grok)
  c4b: n=2  models=2/5 (ChatGPT, Gemini)
  c5a: n=5  models=5/5 (ChatGPT, Claude, Gemini, Grok, DeepSeek)
  c6a: n=4  models=4/5 (ChatGPT, Claude, Gemini, Grok)
  c6b: n=4  models=4/5 (Claude, Gemini, Grok, DeepSeek)
  c6c: n=1  models=1/5 (DeepSeek)
  c7a: n=4  models=4/5 (ChatGPT, Gemini, Grok, DeepSeek)
  c8a: n=4  models=4/5 (ChatGPT, Gemini, Grok, DeepSeek)

================================================================================
6. FULL DOMAIN x MODEL MATRIX (counts)
================================================================================
```

Domain	ChatGPT	Claude	Gemini	Grok	DeepSeek	Total	Models
D1	1	3	1	2	2	9	5/5
D2	1	1	2	1	1	6	5/5
D3	2	2	1	1	2	8	5/5
D4	2	1	1	1	0	5	4/5
D5	1	1	1	1	1	5	5/5
D6	1	2	2	2	2	9	5/5
D7	1	0	1	1	1	4	4/5
D8	1	0	1	1	1	4	4/5

Exported -> analysis_export.json
